# Palladium Nanoparticles/Graphitic Carbon Nitride Nanosheets-Carbon Nanotubes as a Catalytic Amplification Platform for the Selective Determination of 17α-ethinylestradiol in Feedstuffs

**DOI:** 10.1038/s41598-019-50087-2

**Published:** 2019-10-02

**Authors:** Zhi xiang Zheng, Mei Wang, Xue zhao Shi, Chun ming Wang

**Affiliations:** 1 Gansu Province Key Laboratory of Evidence Science Techniques Research and Application, Gansu University of Political Science and Law, 730070 Lanzhou, China; 2 College of Pharmacy, Ningxia Medical University, 750004 Yinchuan, China; 3 College of Chemistry and Chemical Engineering, Lanzhou University, 730001 Lanzhou, China

**Keywords:** Sensors, Electrocatalysis, Electrocatalysis, Two-dimensional materials, Two-dimensional materials

## Abstract

A new kind of nanocomposite, graphitic carbon nitride (g-C_3_N_4_)-carbon nanotubes (CNTs), has been synthesized via solid grinding, and followed by thermal polymerization process of melamine and CNTs. Pd nanoparticles were loaded on the as-prepared nanocomposite by the self-assembly method. The Pd/g-C_3_N_4_-CNTs nanocomposite exhibited excellent electrocatalytic activity toward the oxidation of 17α-ethinylestradiol (EE2), and compared with other detection methods of EE2, such as HPLC, this detection platform does not need the samples for further purification processing. And this detection platform was compared with HPLC, there is no significant difference between two methods, and the accuracy and precision of the determination of EE2 in feedstuff sample by differential pulse voltammetry (DPV) to a satisfactory level. Thus, the Pd/g-C_3_N_4_-CNTs nanocomposite can be used as a signal amplification platform for the detection of EE2 in feedstuffs samples. Under the optimum condition, the current response increased linearly with EE2 concentration from 2.0 × 10^−6^ ~ 1.5 × 10^−4^ M with a detection limit of 5.0 × 10^−7^ M (S/N = 3) by DPV. The Pd/g-C_3_N_4_-CNTs showed good reproducibility and excellent anti-interference ability that the relative standard deviation was 3.3% (n = 5). This strategy may find widespread and promising applications in other sensing systems involving EE2.

## Introduction

Environmental hormone (EH), also known as endocrine disrupting compounds, a kind of exogenous substances, can disrupt the endocrine system of organisms^1^, which have attracted much more attention for causing some diseases even quite low levels (ng/L)^2,3^, such as diabetes, infertility, reproductive disorders, cancer^4,5^. In recent years, an increasing number of EH was found in the water and the soil environment, for example, 17α-Ethinylestradiol (EE2). EE2 is the major constituent of acyeterion, it has positive and negative feedback effect on hypothalamus and pituitary gland, small doses can stimulate gonadotropin secretion, large dose inhibits its secretion, thereby inhibiting the ovulation of the ovary, achieving anti-fertility effect. Its toxicity was dramatically greater than many other similar hormones^6^. In recent years, EE2 was unlawful used as additives to promoting the growth of animals and plants. So, it is necessary to research and develop a simple, rapid, facile and high sensitivity detection method to detect EE2 in foodstuffs. Currently, the high performance liquid chromatography (HPLC), gas chromatography (GC) and electrochemical sensors were used as the detection techniques to determine EE2 in foodstuffs^7–9^. Among these methods, the electrochemical method has received much more attention for its fast response, high sensitivity and selectivity, time-saving and cheap instrument. Unfortunately, as far as we know, the unmodified electrode usually shows poor activity to EE2. Therefore, it is needed to design an electrode material with excellent conductivity, high selectivity, excellent catalytic activity and stable catalysts for the electro-catalytic oxidation of EE2 urgently^9–11^.

Graphitic carbon nitride (g-C_3_N_4_) as a new material, since it was first reported in 1834^12^, since then it has attracted more attention than ever. As a novel non-metal semiconductor material, g-C_3_N_4_ has received more attention due to its chemical durability, adjustable electronic structure and high specific surface area, which appears to provide potential applications as electrochemical sensors^13,14^. The g-C_3_N_4_ planes are composed of tris-triazine (C_6_N_7_) unit connected by a planar tertiary amine groups, so it can easy to capture the target molecules^15^. Nevertheless, bulk g-C_3_N_4_ shows poor conductivity and a high electron hole recombination rate, restricting the electron transportation and electro-catalytic activity^16,17^. So, there have some materials, such as Ag^18^, Fe^19^, Au^20^, Pt^21^, Cu^22^, GO^23^ and CNTs^24^ were used to doped into g-C_3_N_4_ to overcome this defect and improve its electro-catalytic performance. Among these materials, the carbon nanotubes (CNTs) are getting more and more attention because of their special structure, high surface area, unique electrical properties and more surface active sites^25,26^. Recently, many works were reported on the fabrication of g-C_3_N_4_-CNTs composite^27,28^ by a facile sonication/heating method. But few reports about the fabrication of g-C_3_N_4_-CNTs composite by solid grinding method, and used it in EH electrochemical detection. Solid grinding can make g-C_3_N_4_ loading on the CNTs’ surface through electrostatic interaction. During grinding, the precursor of g-C_3_N_4_ diffuses onto the CNTs surface rapidly. Then the precursor polymerized and treated with heat-treatment process in air under proper temperature, leading to C_3_N_4_ -CNTs composites. A significant advantage of solid grinding is that it is easy to operate and the solvent can be avoided. Another advantage is the loading amounts of all introduced precursors can be easily controlled^29^. Our group firstly demonstrated effective loading of melamine on the surface of CNTs by the solid grinding method, and the g-C_3_N_4_-CNTs was prepared by thermos-polymerization which driven by the electrostatic interactions of the π-π stacking between g-C_3_N_4_ and CNTs, and the Pd nanoparticles were self-assemblied on the surface of g-C_3_N_4_-CNTs by reduction of NaPdCl_4_ as precursor using ascorbic acid solution.

In this paper, the g-C_3_N_4_-CNTs nanocomposite was synthesized by direct solid grinding and thermal polymerization, and the Pd/g-C_3_N_4_-CNTs nanocomposite was used as an electrochemical sensor for the determination of EE2 with excellent sensitivity, preferable stability, and reproducibility and antijamming capability in the analysis of samples. The cyclic voltammetry (CV) was employ to study the electrochemical behavior of EE2 on the Pd/g-C_3_N_4_-CNTs/GCE. And difference pulse voltammetry (DPV) was used for the detection of EE2. Compared with other methods, the sensor detection platform does not need the samples for further purification processing. Thus, the Pd/g-C_3_N_4_-CNTs composite as a catalytic amplification platform can provides a useful approach for determining EE2 in feedstuffs.

## Results and Discussion

### Characterization of Pd/g-C3N4-CNTs composite

**Figure 1:**
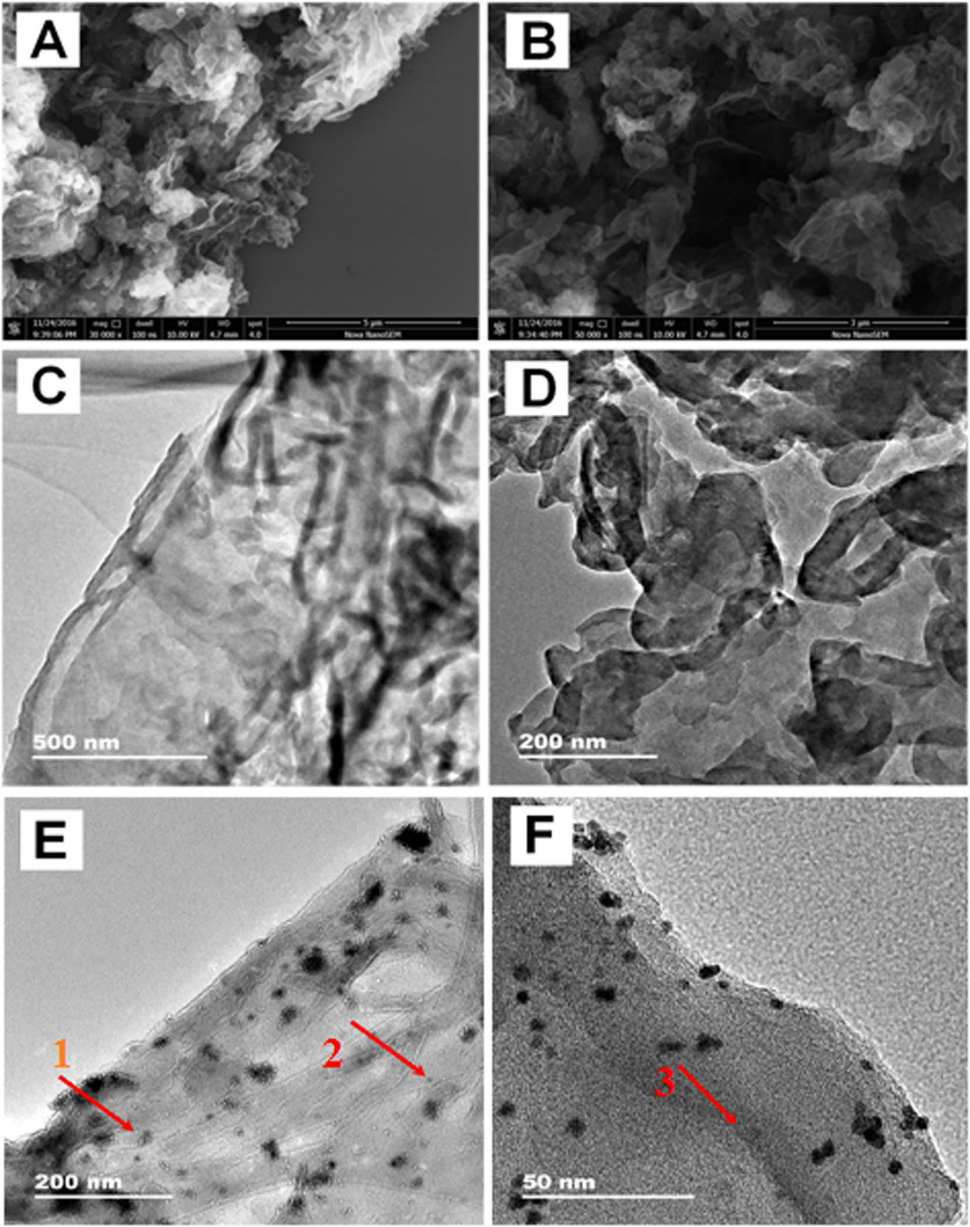
(**A,B**) SEM images of the g-C_3_N_4_-CNTs hybrids; (**C**,**D**) TEM images of the g-C_3_N_4_-CNTs hybrids; (**E**,**F**) TEM images of the Pd/g-C_3_N_4_-CNTs hybrids.

The morphology of the as-prepared g-C_3_N_4_-CNTs and Pd/g-C_3_N_4_-CNTs was investigated via SEM and TEM. Figure 1A,B show the SEM images of the g-C_3_N_4_-CNTs hybrid. Many nanosheets with laminar morphology and two-dimensional (2D) lamellar structure with a ribbon-like structure can be clearly seen which indicated that g-C_3_N_4_ skeleton has no effect on the introduction of CNTs. Additionally; a special structure with CNTs being wrapped by aggregated g-C_3_N_4_ nanosheets can be found, which indicated that g-C_3_N_4_ nanosheets and CNTs have successfully combined together. Similar to SEM results, the TEM of the g-C_3_N_4_-CNTs hybrids also exhibits the flaky structure of the g-C_3_N_4_, tube-like CNTs was well dispersed on the surface of g-C_3_N_4_, indicated that the formation of intimate interfaces between g-C_3_N_4_ and CNTs(Fig. 1C,D). As showed in Fig. 1E, it can be seen that CNTs were wrapped by the g-C_3_N_4_ nanosheets, and Pd nanoparticles very nicely dispersed on the surface or insert into g-C_3_N_4_-CNTs composite forming a sandwich structure (small crystals of Pd were deposited upon the inner layer of g-C_3_N_4_-CNTs composite indicated by red arrows). Figure 1F shows the HRTEM image of Pd/g-C_3_N_4_-CNTs composite. It could be seen that the small crystals of Pd were dispersed on the inner/outer layer of g-C_3_N_4_-CNTs composite similar to low magnification TEM image.

**Figure 2:**
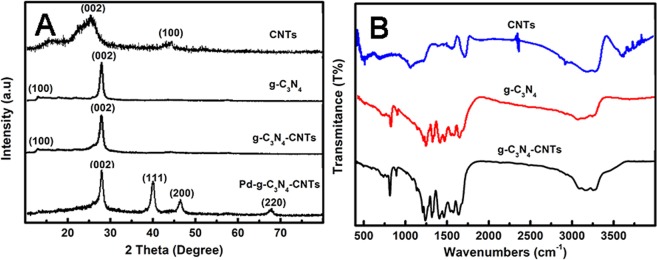
(**A**) XRD patterns of CNTs, bulkg-C_3_N_4_, g-C_3_N_4_-CNTs and Pd/g-C_3_N_4_-CNTs hybrids; (**B**) FTIR spectra of CNTs, bulk g-C_3_N_4_ and g-C_3_N_4_-CNTs hybrids.

The crystal structure of the CNTs, bulk g-C_3_N_4_, g-C_3_N_4_-CNTs and Pd/g-C_3_N_4_-CNTs were analyzed by XRD technique. As shown in Fig. 2A, the CNTs has two distinct diffraction peaks at 26.4° and 45.3°, represents the hexagonal graphite structure, indexed for (002) and (100) directions^30,31^. The bulk g-C_3_N_4_ showed two main diffraction peaks at 13.0° and 27.3°, which should be attributed to in-plane s-triazine units and the stacking of conjugated structure as (100) and (002) directions, in good agreement with the previous reports^32,33^. About the g-C_3_N_4_-CNTs composite, there is no obvious diffraction peaks of CNTs are observed which should be caused by the small amount of CNTs. As for the Pd/g-C_3_N_4_-CNTs/GCE, additional peaks appeared at 40.1°, 47.5° and 68.3°, which are the characteristic diffraction peak of a face-centered-cubic (fcc) crystal phase of the Pd nanoparticles.

The FTIR spectra analysis for CNTs, bulk g-C_3_N_4_, and g-C_3_N_4_-CNTs are shown in Fig. 2B. There is a slight shift of the significant peaks between g-C_3_N_4_ and g-C_3_N_4_-CNTs. The infrared spectral absorption peak at 808 cm^−1^ belongs to the out-of-plane bending mode of triazine ring, which was related to condensed C-N heterocycles^34^. Two strong bands between 900–1800 cm^−1^ range were corresponds to the stretching mode of C-N-C heterocycles and stretching vibrations of aromatic cycles^35^. The broad peak appeared at 3000–3500 cm^−1^ belongs to N-H stretching vibration. The peak appeared at 1570 cm^−1^ is the characteristic of stretching vibration peak of C=C deriving from the inherent structure of CNTs^36^. The peak appeared at 1650 cm^−1^, 1710 cm^−1^, 3400 cm^−1^ belongs to the functionalized CNTs, this corresponding to the carbonyl, carboxyl and hydroxyl functional groups, were identical with those reported in literature^37^. However, the characteristic peaks of the functionalized CNTs could be in the same wavelength range as the C-N triazine heterocycles.

### Electrochemical behaviors of modified electrode

**Figure 3:**
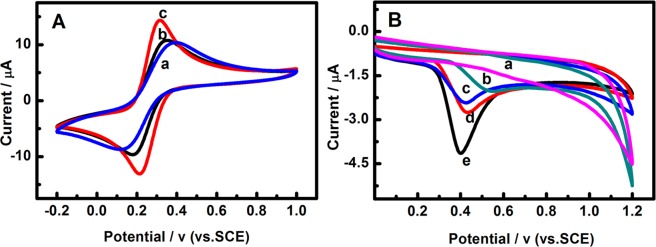
(**A**) Cyclic voltammograms of g-C_3_N_4_/GCE(a), g-C_3_N_4_-CNTs/GCE(b), Pd/g-C_3_N_4_-CNTs/GCE(c) in 5 × 10^−3^ M [Fe(CN)_6_]^3−/4−^ + 0.1 M KCl; (**B**) Cyclic voltammograms of Pd/g-C_3_N_4_-CNTs/GCE(a), GCE(b), g-C_3_N_4_/GCE(c), g-C_3_N_4_-CNTs/GCE (d) and Pd-g-C_3_N_4_-CNTs/GCE (e) in the absence (a) and presence (b, c, d and e) in 0.1 M PBS (pH 10.5) solution containing 0.5 mM EE2 at a scan rate of 70 mV/s.

The electrochemical response signal of [Fe(CN)_6_]^3−/4−^ (1:1) on the modified electrodes were carried out using CV (cyclic voltammetry) techniques. Figure 3A shows the CV behavior of g-C_3_N_4_(a), g-C_3_N_4_-CNTs(b) and Pd/g-C_3_N_4_-CNTs(c) modified GCE in a solution that contains 5.0 mM [Fe(CN)_6_]^3−/4−^ (1:1) and 0.1 M KCl. A pair of redox peaks attributed to the direct redox reaction of [Fe(CN)_6_]^3−/4−^ (1:1) on the g-C_3_N_4_ modified glass carbon electrode(GCE) in the phosphate buffer solution was observed. The peak potential difference (ΔEp = Epa − Epc) of [Fe(CN)_6_]^3−/4−^ (1:1) on the g-C_3_N_4_ modified glass carbon electrode (curve a) is 268 mV. The peak potential difference of g-C_3_N_4_-CNTs/GCE and Pd/g-C_3_N_4_-CNTs/GCE were found to be 170 and 102 mV, respectively (curve b, c). The ΔEp reduction of Pd/g-C_3_N_4_-CNTs/GCE could be attributed to Pd nanoparticles and CNTs which could increase the active surface area, electron transfer kinetics and electronic conductivity. Compared with g-C_3_N_4_/GCE and g-C_3_N_4_-CNTs modified electrode, the redox peaks currents of Pd/g-C_3_N_4_-CNTs increased dramatically, indicated that Pd/g-C_3_N_4_-CNTs could significantly increase the electron transfer rate between [Fe(CN)_6_]^3−/4−^ and electrode surface. The excellent electro-catalytic activity of Pd/g-C_3_N_4_-CNTs may be attributed to the excellent catalytic performance of Pd nanoparticles; the large specific surface area of g-C_3_N_4_-CNTs nanocomposite can provide more active sites, and the excellent electrical conductivity of g-C_3_N_4_-CNTs.

### Cyclic voltammetry behaviors of EE2 on Pd/g-C_3_N_4_-CNTs/GCE

The electrochemical responses of EE2 at bare GCE, g-C_3_N_4_/GCE, g-C_3_N_4_-CNTs/GCE and Pd/g-C_3_N_4_-CNTs/GCE were investigated by CV in 0.1 M PBS buffer (pH = 10.5) containing 0.5 mM EE2 at the scan rate of 70 mV/s. Figure 3B shows the CV behaviors of EE2 on the bare GCE and other modified electrode in the presence (curve b-e) and of absence (curve a) 0.5 mM EE2. There is no response was obtained on the Pd/g-C_3_N_4_-CNTs/GCE (curve a) in the absence of EE2, However, the oxidation peak response was obtained at the bare GCE, g-C_3_N_4_/GCE, g-C_3_N_4_-CNTs/GCE and Pd/g-C_3_N_4_-CNTs/GCE (curve b-e) in the presence of EE2. It is observed that the oxidation peak potential of EE2 at the Pd/g-C_3_N_4_-CNTs/GCE appeared at 0.402 V and the oxidation peak current is 4.15 μA. Compared with the other modified electrode, the oxidation peak current of EE2 at the Pd/g-C_3_N_4_-CNTs/GCE is the largest, which are 2.1, 2.5 and 1.6 times more than the bare GCE, g-C_3_N_4_/GCE and g-C_3_N_4_-CNTs/GCE, respectively. And compared with GCE, the oxidation peak potential moved to negative more than 100 mV. The increase of oxidation currents and the negative of oxidation peak potential should attribute to the Pd nanoparticles and CNTs could enhance the electron transfer efficiency effectively, and the high specific surface area of g-C_3_N_4_-CNTs nanocomposite could provide more surface active sites, and the collaborative catalytic of the Pd nanoparticles, g-C_3_N_4_ and CNTs.

### Effects of scanning rate

**Figure 4:**
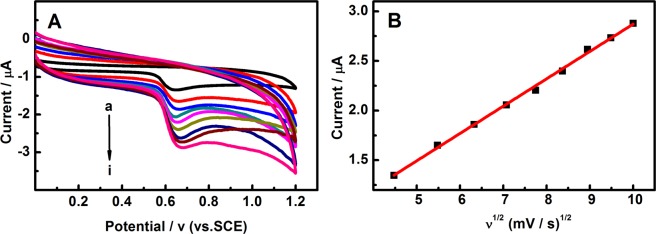
(**A**) CV curves of 0.5 mM EE2 on the Pd/g-C_3_N_4_-CNTs/GCE at different scan rates (a–i): 20, 30, 40, 50, 60, 70, 80, 90 and 100 mV/s; (**B**) The peak currents increased linearly with the square root of scan rate.

The effect of scanning rate of 0.5 mM EE2 at the Pd/g-C_3_N_4_-CNTs/GCE was studied by CV (Fig. 4A). It was found that the oxidation peak current of EE2 was increases with the increase of scanning rate. As shown in Fig. 4B, there is a linear relationship between the increase of the oxidation peak currents and the square root of scanning rate (ν^1/2^) in the range of 20–100 mV/s, which indicated that the electrochemical behavior of EE2 at the Pd/g-C_3_N_4_-CNTs modified electrode is a diffusion-controlled process.

### Effects of solution pH

**Figure 5:**
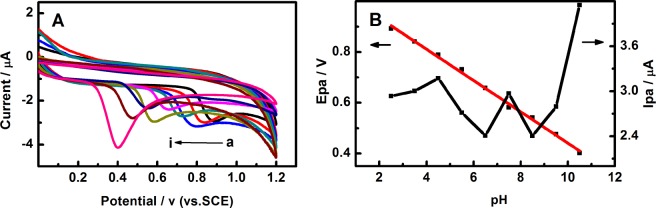
(**A**) CV responses of 0.5 mM EE2 on the Pd/g-C_3_N_4_-CNTs/GCE at different pH range from 2.5 to 10.5 (a–i); (**B**) Effect of solution pH on peak potential and anodic current.

In the electrochemical detection process, the CV behavior of EE2 can be affected by pH of the buffer solution. As shown in Fig. 5A, the influence of pH was studied at the range of 2.0–11.0 by CV. The results indicated that the oxidation peak potentials (Epa) of EE2 shifts forward as the PH increases from 2.5 to 10.5. It indicated that the protons are involved in the electrochemical process. The linear relationship between Epa and pH is Epa(V) = 1.058–0.061 pH, (R = 0.996), the slope is −61 mV/pH for EE2. The slope of EE2 is very close to the theoretical value of −59 mV/pH, which indicated that the electrochemical process of EE2 at Pd/g-C_3_N_4_-CNTs is a two electrons (2e^−^) and two protons (2 H^+^) process^9^.

**Figure 6:**
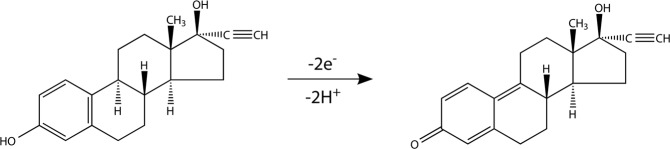
Proposed reaction mechanism for the electrochemical oxidation of 17α-ethinylestradiol.

The effect of pH on the electro-catalytic oxidation of EE2 was shown in Fig. 5B. As can be seen from the diagram, the maximum peak current value appeared at pH 10.5. Consider the detection sensitivity of EE2, the pH = 10.5 of supporting electrolyte was the optimum selection for further electrochemical experiments. According to the literature reports^38,39^ and the experimental facts, the possible reaction mechanism of electrochemical reaction of the E2 at Pd/g-C_3_N_4_-CNTs/GCE was proposed in Fig. 6.

### Electrochemical detection of EE2

**Figure 7:**
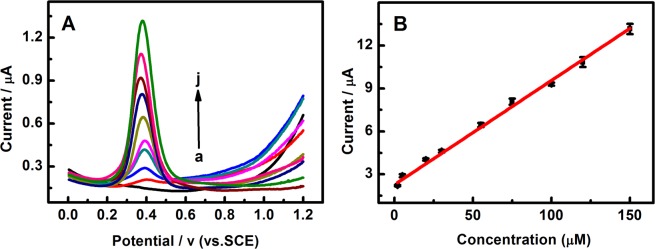
(**A**) DPV curves of EE2 at Pd/g-C_3_N_4_-CNTs/GCE in 0.1 M PBS (pH 10.5) containing different concentrations of EE2 (a-j: 0, 2, 5, 20, 30, 55, 75, 100, 120 and 150 µM); (**B**) The linear relationship between the peak current (Ip_a_) and EE2 concentrations.

Differential pulse voltammetry (DPV) technique was employed to determinate EE2 at Pd/g-C_3_N_4_-CNTs modified electrode. It was evident from Fig. 7, that the anodic peak current (Ipa) of EE2 increased with increasing the concentration of EE2 in the range of 2.0 × 10^−6^ ~ 1.5 × 10^−4^ M. The linear relation of the peak current and concentration of EE2 can be expressed as Ipa (μA) = 2.270 + 0.072C_EE2_ (R = 0.990). The detection limit was 5.0 × 10^−7^ M (S/N = 3).

### Interference, reproducibility and stability studies

**Figure 8:**
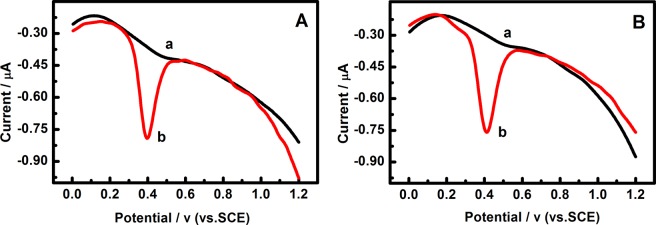
DPV curves of the Pd/g-C_3_N_4_-CNTs/GCE in 0.1 M PBS at pH 10.5 added with chicken feedstuffs solution (**A**); pig feedstuffs solution (**B**) without any EE2 (curve a) and presence of EE2 (curve b).

In this paper, the chicken and pig feedstuffs were soaking in ethanol for 24 h, after that, the soak solution was detected using the Pd/g-C_3_N_4_-CNTs/GCE by the DPV method. So, the interference, reproducibility and stability of Pd/g-C_3_N_4_-CNTs/GCE were studied under the optimal conditions by DPV. We can see the DPV curves of chicken feed solution (A) and pig feed solution (B) in the absence (cures a) and presence (cures b) of EE2 from Fig. 8, which indicated that only an anodic peak of EE2 at 0.40 V was obtained. Similarly, the interference of some common ions, including 100-fold concentrations of Na^+^, Cl^−^, SO_4_^2−^, K^+^ and PO_4_^3−^ was studied. The result indicated that these ions have no influence on the determination of EE2. In other words, the modified electrode has high selectivity and sensitivity for the determination of EE2 in feedstuffs samples. So, the Pd/g-C_3_N_4_-CNTs/GCE is expected to be used for the determination of EE2 in feedstuffs samples.

In the meanwhile, the repeatability and stability of the Pd/g-C_3_N_4_-CNTs modified electrode was studied. In this process, the DPV was employ for the determination of 2.0 μM EE2 in 0.1 M PBS (pH 10.5) solution ten consecutive. The relative standard deviation (RSD) for EE2 was 3.68%, and the current response value remains at 92.3% of its original response after preserved at room temperature for five days, indicated the Pd/g-C_3_N_4_-CNTs modified electrode exhibits remarkable reproducibility and excellent stability.

### Analysis of EE2 in feedstuffs samples

Determination of EE2 in feedstuffs samples was carried out with standard addition method. The chicken and pig feedstuffs were soaking in ethanol for 24 h, after that, the soak solution was detected by DPV. The experimental results were showed in Table 1, the recoveries of EE2 in two feedstuffs sample were 99.1–103.0% and 98.2–106.7%, respectively. Table 2 is the detection index of this sensor compared with other sensors. It indicated that this method have wide detection range than those previously reported.

**Table 1:** The determination results of EE2 in feedstuffs samples (n = 5).

Samples	Added EE2 (μM)	Detected EE2 (μM)	RSD (%)	Recover (%)	Bias (%)
Chicken feedstuffs solution	30.00	30.90	1.81	103.0	+3.0
	55.00	54.51	2.95	99.1	−0.9
	75.00	75.84	2.14	101.1	+1.1
Pig feedstuffs solution	10.00	10.67	3.30	106.7	+6.7
	20.00	19.63	1.64	98.2	−1.8
	30.00	31.15	1.37	103.8	+3.8

**Table 2:** Performance comparison of the proposal sensor for EE2 detection with other sensors.

Sample	Electrode	Method	Linger rage (μM)	Detection limit (μM)	Ref.
EE2	(Chi/CNT)_3_/FTO	DPV	0.05–20	0.09	^9^
EE2	CPB/CPE	DPV	0.05–20	0.03	^10^
E2	BPIDS/GCE	DPV	0.1–10	0.05	^41^
E1	Pt/MWCNTs	DPV	2–50	0.84	^42^
EE2	Pd/g-C_3_N_4_-CNTs	DPV	2–150	0.50	This work

^a^(Chi/CNT)_3_/FTO: Chitosan/multi-walled carbon nanotubes/fluorine doped tin oxide.

^b^CPB/CPE: Cetyl pyridine bromide/ carbon paste electrode.

^c^BPIDS/GCE: 1-butyl-3-[3-(N-pyrrolyl)propyl]imidazolium dodecyl sulfate/glassy carbon electrode.

### Significant difference (SD) of detection EE2 by DPV and HPLC

So as to account for feasibility of detection EE2 by this sensor, Under the same conditions, the high performance liquid chromatography (HPLC) was employing for the detection of EE2 in the sample. After that, the statistical product and service solutions (SPSS) 17.0 was used to compare the significant difference **(SD)** of two means. Recoveries were calculated from the average recoveries of three replicate samples. The determination results are compared with the electrochemical method that was shown in Table S1. The T-test of SPSS statistical approach was using to contrast the determination results of EE2 by DPV and HPLC which was shown in Table S2. The P-value measured by the F-test was 0.381, and the P-value was greater than 0.05, indicating that the homogeneity of variance. The P-value measured by the T-test was 0.409 and the P-value was greater than 0.05. The result indicated that there is no significant difference **(SD)** between two methods. The accuracy and precision of the determination of EE2 in feedstuff sample by DPV method to a satisfactory level. So, as a new type of catalytic material, Pd/g-C_3_N_4_-CNTs can be used for the detection of EE2 in feedstuff sample.

## Conclusions

In this paper, the g-C_3_N_4_-CNTs composite was prepared by directly solid grinding and thermal polymerization in air. After that, the Pd nanoparticles modified g-C_3_N_4_-CNTs was prepared by self-assembly method. The Pd/g-C_3_N_4_-CNTs was used to determine EE2 in practice samples. The fabricated sensor showed higher stability, good repeatability and antijamming capability in real samples. And compared with other methods, the sensor detection platform does not need the samples for further purification processing.

## Methods

The g-C_3_N_4_-CNTs composite was synthesized by directly solid grinding and thermal polymerization in air, and the Pd/g-C_3_N_4_-CNTs composite was prepared by the self-assembly method^40^, and the Pd/g-C_3_N_4_-CNTs modified electrode was prepared by the conventional methods. The reagents, apparatus, and the detailed processes of the material’s preparation in this paper were showed in the supporting information.

## Supplementary information

Palladium Nanoparticles/Graphitic Carbon Nitride Nanosheets-Carbon Nanotubes as a Catalytic Amplification Platform for the Selective Determination of 17α-ethinylestradiol in Feedstuffs

## Data Availability

The datasets generated during and/or analyzed during the current study are available from the corresponding authors on request.
