# Supplementary material for: Palladium Nanoparticles/Graphitic Carbon Nitride Nanosheets-Carbon Nanotubes as a Catalytic Amplification Platform for the Selective Determination of 17α-ethinylestradiol in Feedstuffs
Source: Sci Rep. 2019 Oct 2;9:14162. doi: 10.1038/s41598-019-50087-2 (PMC6775042; doi:10.1038/s41598-019-50087-2)
Supplement: Supplementary file 1 — Palladium Nanoparticles/Graphitic Carbon Nitride Nanosheets-Carbon Nanotubes as a Catalytic Amplification Platform for the Selective Determination of 17α-ethinylestradiol in Feedstuffs [file 41598_2019_50087_MOESM1_ESM.pdf]

Palladium Nanoparticles/Graphitic Carbon Nitride Nanosheets-Carbon Nanotubes as a Catalytic Amplification Platform for the Selective Determination of 17α-ethinylestradiol in Feedstuffs

Zhi xiang Zheng[a,b*], Mei Wang[c], Xue zhao Shi[d], Chun ming Wang[d*]

a Key Laboratory of Evidence Science Techniques Research and Application, Gansu Province, 730070, Lanzhou, China

b Gansu Institute of Political Science and Law, 730070, Lanzhou, China

c College of Pharmacy, Ningxia Medical University, 750004, Yinchuan, China

d College of Chemistry and Chemical Engineering, Lanzhou University, 730001, Lanzhou, China

Supporting information

1. Significant difference of detection EE2 by DPV and HPLC

So as to account for the feasibility of the sensor for the detection of EE2，under the same conditions, the high performance liquid chromatography (HPLC) was employed for the detection of EE2 in the feedstuff sample. After that，the statistical product and service solutions (SPSS) 17.0 was used to compare the significant difference of two means. The prepared samples were analyzed on a TOSOH TSKgel-ODS column（250 mm×4.6 mm I.D, 5 μm）from Knauer series Smartline pump (Berlin, Germany). Mobile phase was nitrile/water (45:55) mixture. The injection volume was 20 μL. Flow rate is 1.0 mL/min. Test wavelength is 280 nm. The process of sample treatment：5.0 g chicken feed samples was added to 25 mL

Corresponding author. fax: +86 9317601409
E-mail address: dqzhenghao@163.com(Z.X. Zheng) , wangcm@lzu.edu.cn(C. M. Wang)

methanol and soaking for 24 h. after that, centrifugation technology was used to treat the sample, the supernatant was filtered by 0.22 μm membrane for backup. The regression equations of EE2 is y=118349x+46819(R= 0.993), and the liner range is 3.7~59.2 mg/L；Accuracy measurement：Divide 5.0 g chicken feed sample into six portions，after that, 55μL of EE2 standard solution was added to the sample at room temperature. Recoveries were calculated from the average recoveries of three replicate samples. The determination results of HPLC were compared with the electrochemical method that was shown in Table Ⅲ.

Table Ⅰ Determination results of the feedstuffs samples of EE2

Samples	Method	Added	Added (mg/L)	Detected(mg/L)	Recover (%)	RSD (%)
Chicken feed solution	DPV	EE2	16.28	16.13	99.10	2.95
	HPLC	EE2	16.28	16.56	101.71	2.00

The T-test of SPSS statistical approach was used to contrast the determination results of EE2 by DPV and HPLC shown in TableⅣ. That the p-value measured by the F-test was 0.381, and the p-value was greater than 0.05, indicated the homogeneity of variance. The p-value measured by the T-test was 0.409 and the p-value was greater than 0.05. It was indicated that there is no significant difference between the two methods. The accuracy and precision of the determination of EE2 in feedstuff sample by DPV method can get to a satisfactory level. As a new type of catalytic material, Pd/g-C_3N_4-CNTs nanocomposite can be used as a kind of sensor for the detection of EE2 in feedstuff sample.

Table Ⅱ DPV and HPLC method of F and T test

EE2	Levene test of variance equation		T-test of the mean value equation						
								confidence interval 95%	
	F	Siq.	T	df	Siq.（both	Mean difference	Standard error	lower limit	upper limit

						sides）				
estimated value	Equal Variances Assumed	0.895	0.381	-0.888	6.0	0.409	-0.43267	0.48733	-1.62512	0.75979
	Equal variances not assumed			-0.983	5.686	0.366	-0.43267	0.44028	-1.52456	0.65922

2. The TEM images of g-C_3N_4, g-C_3N_4-CNTs Pd/g-C_3N_4-CNTs

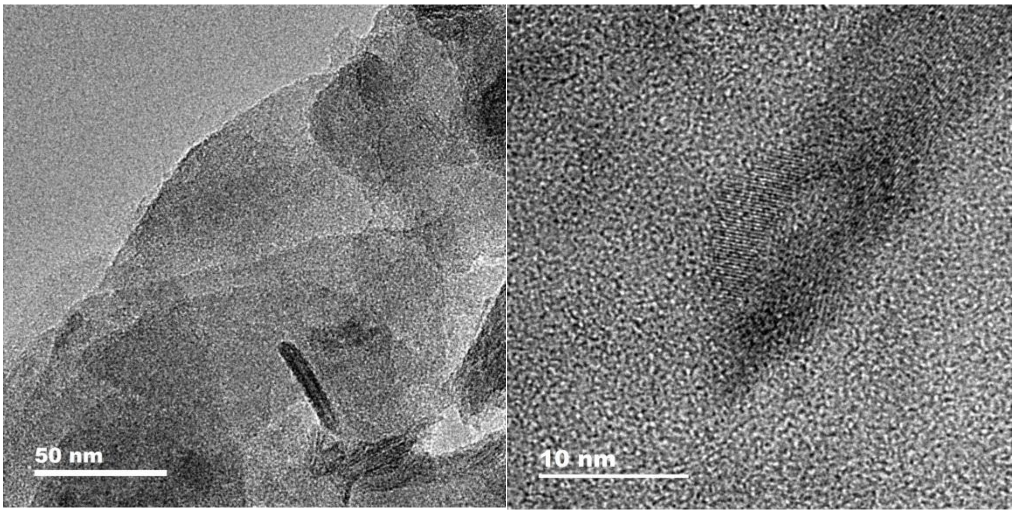

Fig. S1 TEM images of g-C_3N_4, g-C_3N_4-CNTs with higher resolution

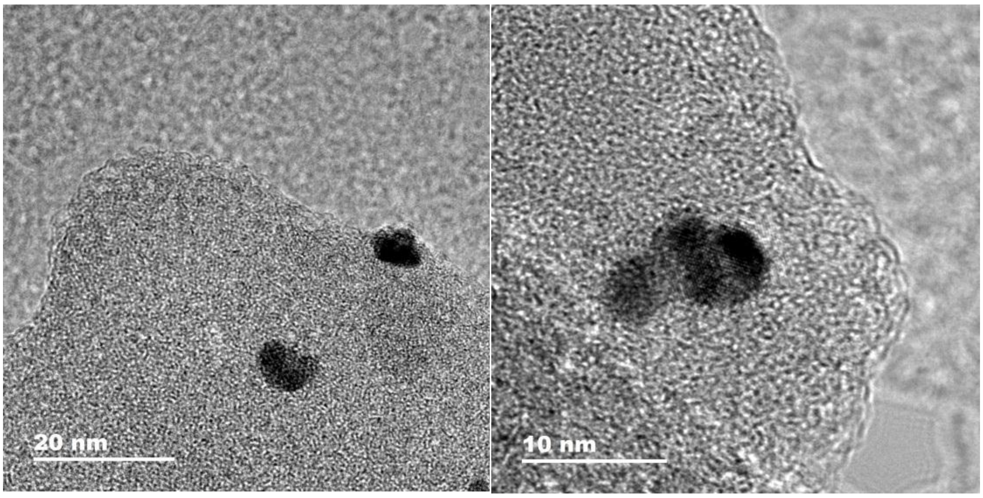

Fig. S2 TEM images of Pd/g-C_3N_4-CNTs hybrids with higher resolution

3. Methods

3.1. Reagents and apparatus

Ethinylestradiol (EE2) and ascorbic acid (AA) was purchased from aladdin Co., Ltd. All reagents were of analytical grade and used without further purification. The PBS buffer solution (0.1 M, pH 10.5) was prepared from Na_2HPO_4 and NaH_2PO_4 and its pH was adjusted to 10.5 using 1.0 M NaOH. All solutions were prepared with ultra-pure water.

The surface morphology of g-C_3N_4-CNTs was investigated via scanning electron microscopy (SEM, JSM-6700F Japan) and transmission electron microscope (TEM, Tecnai G2 F30, FEI, USA). The crystalline structures of the samples were measured by X-ray diffraction (XRD) experiments on an X-ray diffraction analyzer (XRD, Rigaku D/max-2400, Cu Kα radiation, λ=0.15418 nm). Fourier transform infrared (FTIR) spectra were recorded on a TENSOR37 FT-IR spectrometer (Germany). The electrochemical experiments were carried out on a CHI660E electrochemical work station (Shanghai Chenhua Instrument Company, China), with a three-electrode configuration that the Pd/g-C_3N_4-CNTs/GCE, a platinum wire and a saturated calomel electrode (SCE) to be the working electrode, counter electrode and reference electrode, respectively. Prior to each experiment, the solution was deoxygenated with nitrogen and kept in nitrogen atmosphere at room temperature.

3.2. Preparation of Pd/g-C_3N_4-CNTs

The g-C_3N_4-CNTs composite was synthesized by directly solid grinding and thermal polymerization in air (Fig. 9). In a typical process, 2.50 g of melamine and 0.12 g of

CNTs mixture were grinding for 2 h at room temperature, and then put into alumina crucible with a tinfoil cover. After that, the mixture was polymerization by heating from room temperature to 520 ℃ at a heating rate of 5 ℃/min, keeping the temperature at 520 ℃ for 4 h, the g-C_3N_4-CNTs composite was acquired after natural cooling.

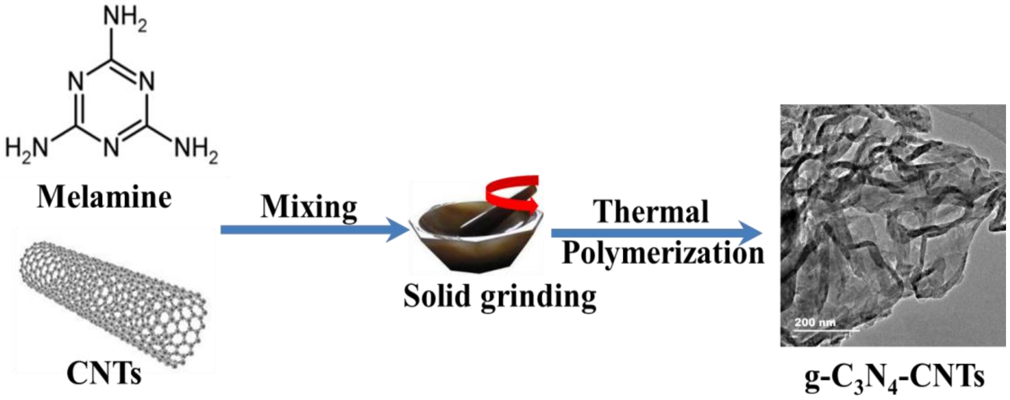

Fig. S3 Synthesis of g-C_3N_4-CNTs via solid grinding of melamine and CNTs followed by thermal polymerization

The Pd/g-C_3N_4-CNTs composite was prepared by the self-assembly method[30]. Briefly, Na_2PdCl_4 solution (0.6 mL 20.0 mM) and HCl solution (0.1 mL, 6.0 M) were mixed into g-C_3N_4-CNTs (20.0 mL, 0.5 mg/mL). The mixed solution was stirred for 5 hours, and ascorbic acid solution (AA, 4.0 mL, 0.1 M) was added to the above solution under stirring. Then, the above solution was kept in an oil bath for 4 hours at 95 ℃. Finally, the nanocomposite was separated from the solution by centrifugation at 14000 rpm for 20 min and the un-reacted species were removed by multiple washing steps with ultra-pure water and ethanol.

3.3. Preparation of Pd/g-C_3N_4-CNTs modified electrode

The modified electrode was prepared by the conventional method [1]. Briefly, A glassy carbon electrode was polished with alumina suspensions (1.0 and 0.05μm), and washed with ethanol/deionized water (1:1, v/v) for 5 min under an ultrasonic bath. Then, the electrode was dried at room temperature. 1.0 mg of Pd/g-C_3N_4-CNTs hybrid was added to 1.0 mL of ultra-pure water and sonicated for 0.5 h. Approximately 5.0 μL of the Pd/g-C_3N_4-CNTs was dropped onto the electrode surface, and then dried at room temperature. The modified electrode was denoted as Pd/g-C_3N_4-CNTs/GCE.

References

[1] Wang M., Zheng Z.X., Liu J.J. & Wang C.M., Pt-Pd bimetallic nanoparticles decorated nanoporous graphene as a catalytic amplification platform for electrochemical detection of Xanthine, Electroanalysis. 29, 1258-1266 (2017).
